# Laparoscopic radical proximal gastrectomy with double flap technique for early proximal gastric cancer: a prospective, phase II study

**DOI:** 10.3389/fonc.2026.1830984

**Published:** 2026-04-29

**Authors:** Shengning Zhou, Guangyu Zhong, Hongming Li, Jintao Zeng, Yang Chen, Fanghai Han, Jianan Tan, Yequan Xie

**Affiliations:** 1 Department of Gastrointestinal Surgery, The Affiliated Guangdong Second Provincial General Hospital of Jinan University, Guangzhou, Guangdong, China; 2 Department of Gastrointestinal Surgery, Guangdong Provincial Hospital of Traditional Chinese Medicine, Guangzhou, Guangdong, China; 3 Department of Gastrointestinal Surgery, Sun Yat-Sen Memorial Hospital of Sun Yat-Sen University, Guangzhou, Guangdong, China

**Keywords:** double flap technique, early gastric cancer, laparoscopic proximal gastrectomy, quality of life, reflux esophagitis

## Abstract

**Background:**

Proximal gastrectomy preserves gastric function but is limited by postoperative reflux. The double flap technique (DFT) offers an anti-reflux reconstruction, yet prospective evidence in China is lacking.

**Methods:**

This prospective, single-center phase II study evaluated the safety and efficacy of laparoscopic proximal gastrectomy with DFT (LPG-DFT) for early proximal gastric cancer. Primary outcome was reflux esophagitis at 12 months; secondary outcomes included perioperative results, nutrition, and quality of life (QoL).

**Results:**

Twenty patients underwent LPG-DFT without intraoperative complications or conversion. All achieved R0 resection, with a mean of 27.3 ± 8.9 lymph nodes harvested. No patient developed Los Angeles grade B or higher reflux at 12 months. Nutritional parameters showed only slight, nonsignificant declines. QoL scores decreased temporarily at 3 months but returned to baseline by 12 months. No major complications or mortality occurred.

**Conclusion:**

LPG-DFT is safe, effectively prevents reflux, and preserves nutrition and QoL, supporting its use in early proximal gastric cancer.

## Background

Gastric cancer is the fifth most common malignant tumor in the world, and its mortality rate ranks fourth (1, 2). In China, the incidence rate (47.9 per 100000) and mortality rate (37.4 per 100000) of gastric cancer rank 3rd among all malignant tumors (3). Although the total incidence of gastric cancer has been steadily declining worldwide, the trend for proximal gastric cancer is rising (4–7). Thus, it is still necessary to choose appropriate surgical resection and reconstruction methods to improve the prognosis of patients with proximal gastric cancer (8).

The 6th edition of the Japanese Guidelines for the Treatment of Gastric Cancer (9) stated that the standard surgical procedure for upper advanced gastric cancer is total gastrectomy, and for early proximal gastric cancer, radical total gastrectomy or radical proximal gastrectomy can be selected. Studies have shown that there is no statistically significant difference in postoperative overall survival rate between patients who underwent total gastrectomy and those who underwent proximal gastrectomy for early upper gastric cancer (10–17). However, no consensus has been reached on how to perform digestive tract reconstruction in PG surgery, and there is no high-level evidence-based medical evidence. Compared with total gastrectomy, proximal gastrectomy (PG) has certain advantages in postoperative gastrointestinal function and nutritional status, and increases the feasibility of postoperative endoscopic examination in patients. However, the quality of life of patients with reflux esophagitis after proximal gastrectomy can be seriously affected if only a simple esophagogastric stump anastomosis is performed (18, 19). According to Japanese reports, the incidence of reflux esophagitis after simple esophagus-residual stomach anastomosis reached 62%, and the incidence of reflux esophagitis, anastomotic leakage and anastomotic stenosis were all high (18). Although it benefited from nutrition, it seriously affected the quality of life, and the short-term effect was not as good as radical total gastrectomy. For this reason, most surgeons at home and abroad still choose radical total gastrectomy for early proximal gastric cancer. If it can reduce the incidence of reflux esophagitis, proximal gastrectomy will be an optional treatment for early proximal gastric cancer, which may significantly improve the quality of life and nutritional indexes compared with total gastrectomy.

The double flap technique (DFT) is an esophagogastric reconstruction method after PG used by Kamikawa in 1998 for proximal gastric ulcers. This method has been used in LPG for proximal gastric cancer since 2016 (20, 21). According to previous reports, LPG with DFT (LPG-DFT) was superior to laparoscopic total gastrectomy with Roux-en-Y reconstruction (LTG-RY) in postoperative nutritional maintenance and the incidence of reflux esophagitis and can be the first option for proximal early gastric cancer (13, 14). Under this premise, we conducted a prospective one-arm study to further verify the safety and efficacy of LPG-DFT.

## Materials and methods

### Study design

This is a prospective, single-center, single-arm, phase II clinical trial. The primary outcome was incidences of reflux esophagitis and anastomotic stenosis 12 months after operation, and the secondary outcomes were nutrition indicators, operative time, number of harvested lymph nodes, R0 resection rate, intraoperative blood loss, postoperative quality of life (Qol), morbidity, and mortality. The study was reviewed and approved by the Ethics Committee of Sun Yat-sen Memorial Hospital, Sun Yat-sen University, and registered in the Chinese Clinical Trial Registry (http://www.chictr.org.cn. Registration number: ChiCTR2000037823).

### Participants

Eligible criteria were as follow:

1. Aged 20–80 years; 2. Informed consent; 3. The proximal 1/3 primary gastric lesion was pathologically diagnosed as gastric adenocarcinoma by endoscopic biopsy; 4. Preoperative staging was T1N0-1M0 or T2N0M0 (according to AJCC-8th TNM tumor staging (22)); 5. Preoperative ASA score was I-III; 6. Patients with cardiopulmonary function can tolerate laparoscopic surgery; 7.Scheduled for laparoscopic proximal gastrectomy with D1+/D2 lymph node dissection (per 2021 Japanese Gastric Cancer Treatment Guidelines 6th edition (9)), potentially achieving R0 resection.

Exclusion criteria were as follow:1. The patient has a history of upper abdominal surgery and is not suitable for laparoscopic surgery. 2. The intraoperative anastomosis of residual esophagus and stomach is too large, so the reconstruction method needs to be changed. 3. The tumor invaded the esophagus by more than 3cm of dentate line; 4. Has a serious mental illness; 5. Pregnant women; 6. A history of continuous systemic corticosteroid or immunosuppressive drug therapy within 1 month. 7. history of neoadjuvant therapy; 8. under emergency surgery; 9. requirement for simultaneous surgery due to complications with other diseases; 10.with other malignant diseases or have suffered from other malignant diseases within 5 years.

Patients should be eliminated with followed criteria: after enrollment, severe comorbidities (intolerability of surgery or anesthesia) occurred before surgery, compliance to the protocol could not be achieved expectedly; the patient voluntarily requests to withdraw or discontinue treatment for personal reasons at any stage after entering the study; proved to have administered treatment in violation of the study protocol.

### Team of surgeons

All the surgeries were performed by a surgical team, with Professor Han Fanghai as the chief surgeon, Professor Yang Bin as the assistant, and Dr. Zhou Shengning as the scope holder. Prior to this clinical trial, the surgical team had done more than 300 cases of laparoscopic radical gastrectomy; at the time of the study, more than 80 cases of laparoscopic radical gastrectomy were being performed annually.

### Preoperative assessment

Preoperative assessment: All baseline data were collected on the day of hospital admission, generally within one week before surgery. Baseline evaluations included age, sex, height, body weight, body mass index (BMI), comorbidities, preoperative blood tests (including routine blood examination, biochemical analysis, and tumor markers), endoscopy, and computed tomography (CT).

### Surgical procedure

General anesthesia was needed. The pressure of pneumoperitoneum was maintain at 12–13 mmHg. The number of trocar was no more than 5 and assisted incision can only be 1 and its length must be less than 10 cm.

**Figure 1:**
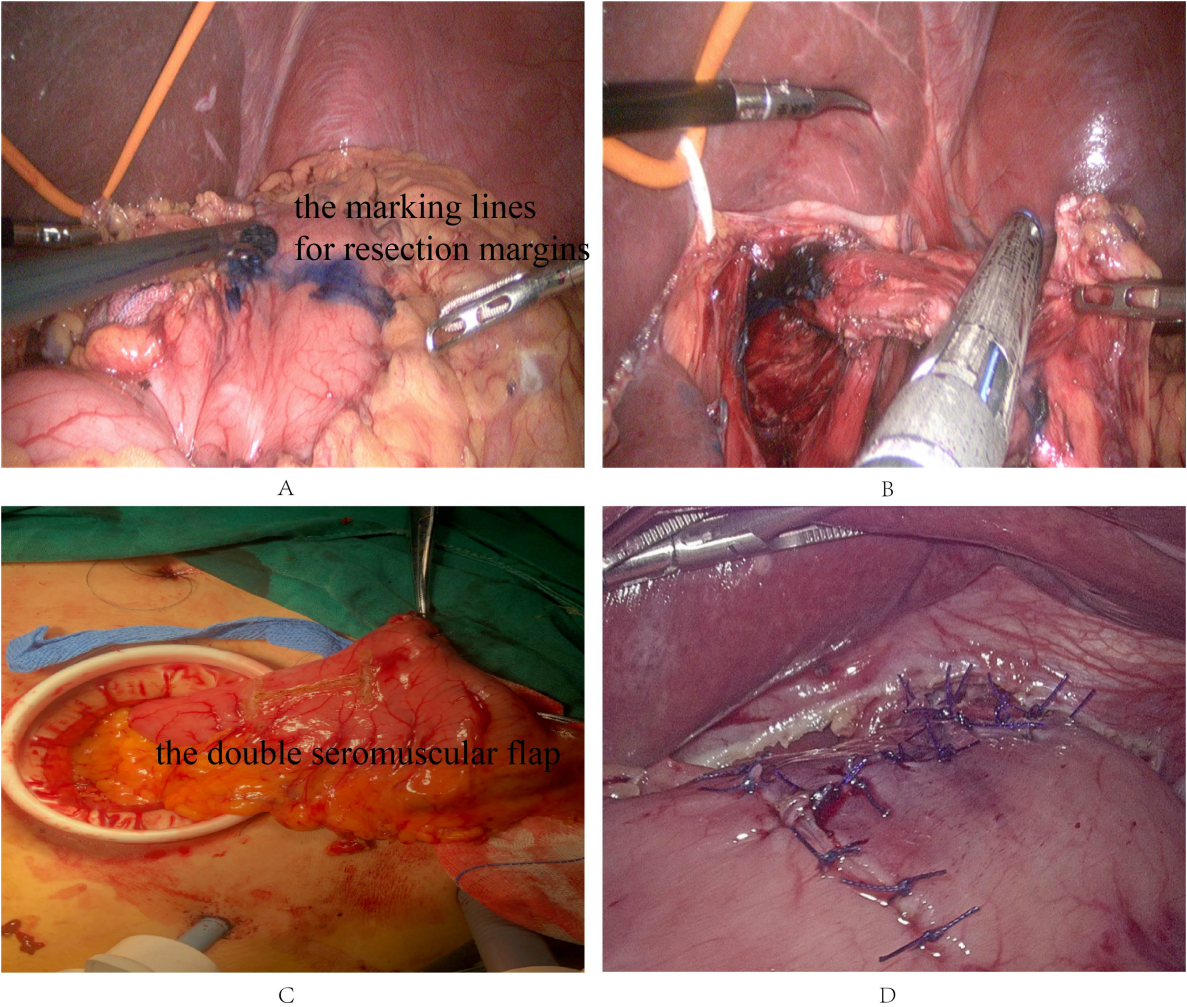
Intraoperative situation of double flap technique. **(A, B)** Determine the proximal and distal resection margins of the stomach. **(C)** Create a seromuscular flap on the anterior wall of the distal remnant stomach extracorporeally. **(D)** Perform anastomosis between the esophagus and the remnant stomach, and finally, completely cover the anastomosis with the double seromuscular flap.

Laparoscopic radical proximal gastrectomy with D1+ or D2 lymphadenectomy was performed, D1+ (No.s 1, 2, 3a, 4sa, 4sb, 7, 8a, 9 and 11p groups) for stage T1N0 while D2 (No.s 1, 2, 3a, 4sa, 4sb, 7, 8a, 9, 11p and 11d groups) for stages T1N1 and T2N0 according to the criteria of the latest Japanese Gastric Cancer Treatment Guidelines (9). Omentectomy was performed partially. Proximal and distal margins are at least 2 cm in cT1 patients and 3 cm in cT2 patients, respectively, negative frozen pathological examination is also needed in all cases. Esophagogastric reconstruction was performed by the double-flap technique (21). The double seromuscular flap (2.5 cm in width and 3.5 cm in height) was created on the anterior wall of the distal remnant stomach using electrocautery. An opening was made at the lower end of the mucosal window, and the upper end of the gastric mucosal window was fixed to the posterior wall of the esophageal stump, approximately 5 cm from the transection margin. Subsequently, the esophagus was anastomosed to the exposed gastric mucosa, and finally, the anastomosis was completely covered with the double seromuscular flap. The double flaps are allowed to be created extracorporeally, and esophagogastric reconstruction is done intracorporeally (**Figure 1**).

Intraoperative complications such as massive bleeding or accident injury of other organs occur and it is difficult to control under laparoscopy, conversion to open surgery must be actively performed.

### Surgical quality control

Electronic gastroscopic examination is used for intraoperative tumor localization and quality control of esophagogastric anastomosis. An air leak test is done after esophagogastric reconstruction.

### Postoperative management

The gastric tube is removed on the first day after operation unless gastric fluid flow is greater than 200ml per day. The abdominal drainage tube was removed after 3 days postoperatively, with drainage fluid clear and is less than 100ml per day. Postoperative contrast examination was performed on postoperative day 4 to assess the presence of anastomotic stenosis or leakage.

A liquid diet is given after the gastric tube was removed, and a semi-liquid diet is given after the patient was able to tolerate the liquid diet for 1 day. Patient would discharge when they can tolerate soft food, walk from bed to toilet by themselves and take care of themselves in basic life without complications. Postoperative complications were estimated comprehensively by investigators via symptoms, signs, laboratory tests and imaging examinations. The severity of the morbidity was assessed according to the Clavien-Dindo classification.

### Outcomes

#### Primary outcome

The primary endpoint of this trial is the proportion of patients with reflux esophagitis within 12 months postoperatively. Reflux esophagitis is graded according to the Los Angeles (LA) Classification at the 1-year follow-up endoscopy. Grade A: One (or more) mucosal break no longer than 5 mm that does not extend between the tops of two mucosal folds. Grade B: One (or more) mucosal break more than 5 mm long that does not extend between the tops of two mucosal folds. Grade C: One (or more) mucosal break that is continuous between the tops of two or more mucosal folds but which involves less than 75% of the circumference. Grade D: One (or more) mucosal break that involves at least 75% of the esophageal circumference (23). The Los Angeles grade of more than B represents severe reflux esophagitis.

#### Secondary outcomes

Secondary outcomes encompass intraoperative metrics (total operation time, reconstruction duration, estimated blood loss, lymph node dissection extent), postoperative recovery indicators (diet initiation, first flatus, gastric tube duration, hospitalization length, rehospitalization rate), and pain assessment via VAS (24) at 24 hours. Pathological outcomes include positive lymph nodes, R0 resection rate, and tumor size from postoperative examination. Quality of life (QoL) is evaluated using EORTC QLQ-C30 (25) and STO22 (26) questionnaires at baseline, 3, and 12-month follow-ups. Nutrition status is monitored through weight, BMI, protein levels, albumin, prealbumin, hemoglobin, and VitB12. Endoscopic examination was also performed to assess the presence of anastomotic stenosis. Postoperative morbidity is categorized as early (≤30 days) or late (>30 days) and classified by Clavien-Dindo standards (27). Mortality records include date and cause of death. All data collection adheres to predefined timelines and diagnostic criteria.

### Blinding

The trial is a single-center, single-arm, prospective clinical trial, so the surgical procedures and postoperative intervention will not blind either the care providers, investigators, or participants.

### Sample size calculation

**Figure 2:**
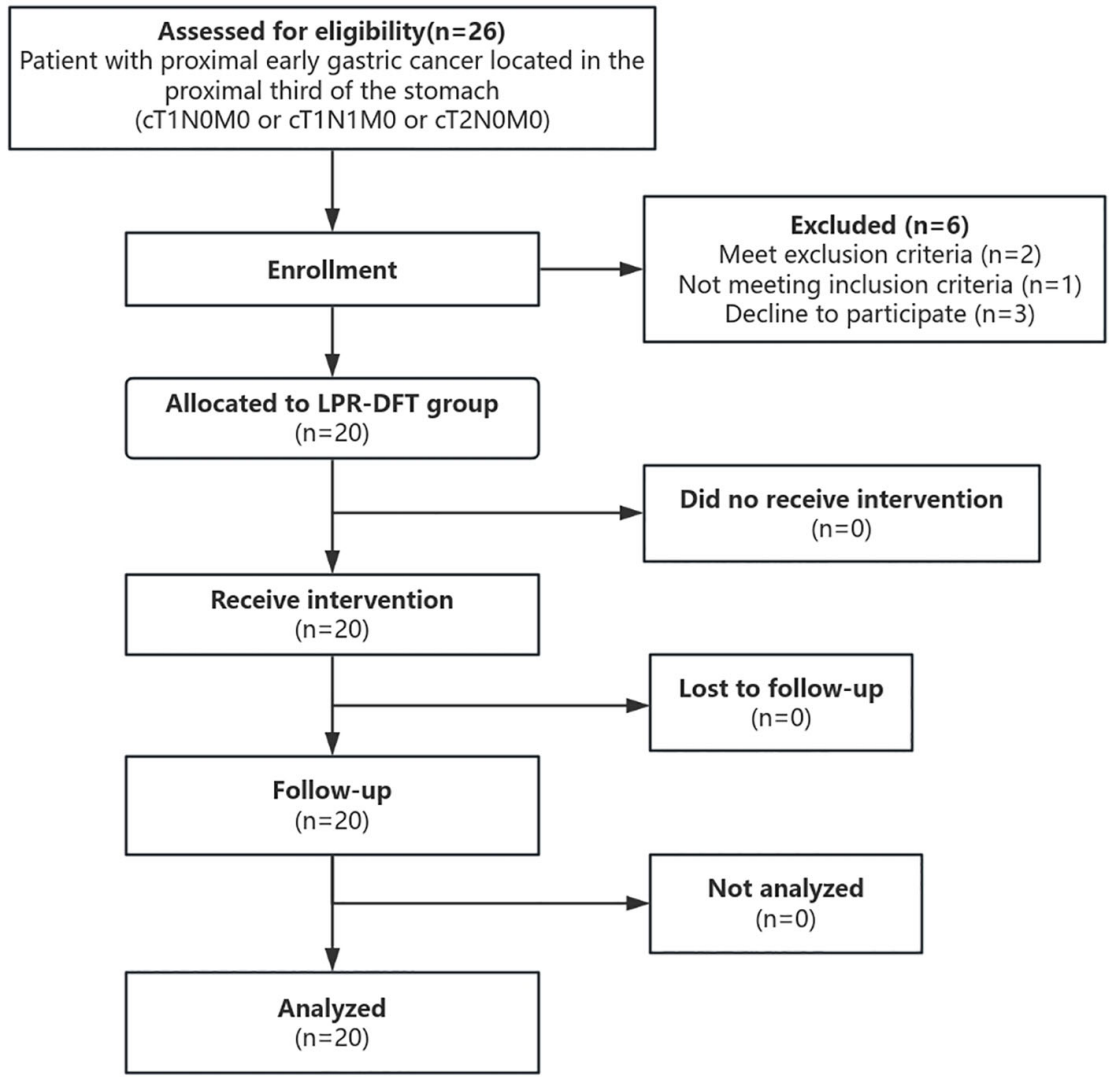
Flowchart of this study. Through enrollment, 20 patients who met the inclusion and exclusion criteria were assigned to the LPG-DFT group and accepted follow-up and analysis.

This clinical trial aimed to evaluate the 12-month incidence of reflux esophagitis (Los Angeles grade B or more) after LPG-DFT for proximal early gastric cancer. Based on prior reports, the estimated rate ranged from 2.0% to 4.2% (14, 21, 28), leading us to conservatively set the expected rate at 3.0% for sample size calculation. However, due to practical constraints, the study was conducted with 20 participants (**Figure 2**). While this sample size limits statistical power compared to the initial requirement, it remains valid as a preliminary exploratory study, providing valuable descriptive data on postoperative outcomes.

### Statistical analysis

Statistical analysis was carried out by software SPSS 25.0 for Windows. Full Analysis Set (FAS) includes all patients who have undergone operation, while Per-Protocal Set (PPS) includes patients in FAS who have received gastroscoppic examination 12 months after surgery. Frequency N and percentage % will be used for descriptive statistics of classified data, while Mean, Standard deviation (SD), Median, Minimum and Maximum will be used for continuous data. Continuous variables measured at different time points were compared using paired t-tests. ANOVA, chi-square or Fisher’s exact test were used for univariate analysis, and logistic regression was used for multivariate analysis. A value of P < 0.05 was considered statistically significant.

## Results

### Patient clinical characteristics

Between June 1, 2019 and December 24, 2022 at Sun Yat-sen Memorial Hospital, 26 patients were initially enrolled in the study, with 20 ultimately undergoing laparoscopic radical proximal gastrectomy with double flap technique (**Figure 2**). The cohort consisted of 12 male and 8 female patients with a mean age of 59.3 ± 6.2 years and mean BMI of 23.0 ± 2.4 kg/m². Notably, none of the cases required conversion to open surgery. Detailed clinical and pathological characteristics, including ASA score, ECOG performance status, preoperative CEA levels, clinical staging, and medical history, are presented in **Table 1**.

**Table 1:** Patient demographics and clinical characteristics.

Indicators	Patients (*n* = 20)
Age(years)	59.3 ± 6.2
Male	12 (60%)
BMI (kg/m^2^)	23.0 ± 2.4
ASA score	
1	11 (55%)
2	8 (40%)
3	1 (5%)
ECOG	
0	19(95%)
1	1(5%)
Preoperative CEA (ng/ml)	3.1 ± 1.6
Median	2.81 [0.82-6.88]
≤5.0	18 (90%)
>5.0	2 (10%)
Clinical stage	
T1N0	5 (25%)
T1N1	7 (35%)
T2N0	8 (40%)
Coronary heart disease	4 (20%)
Hypertension	7 (35%)
Diabetes	8 (40%)

### Postoperative pathological and clinical outcomes

**Figure 3:**
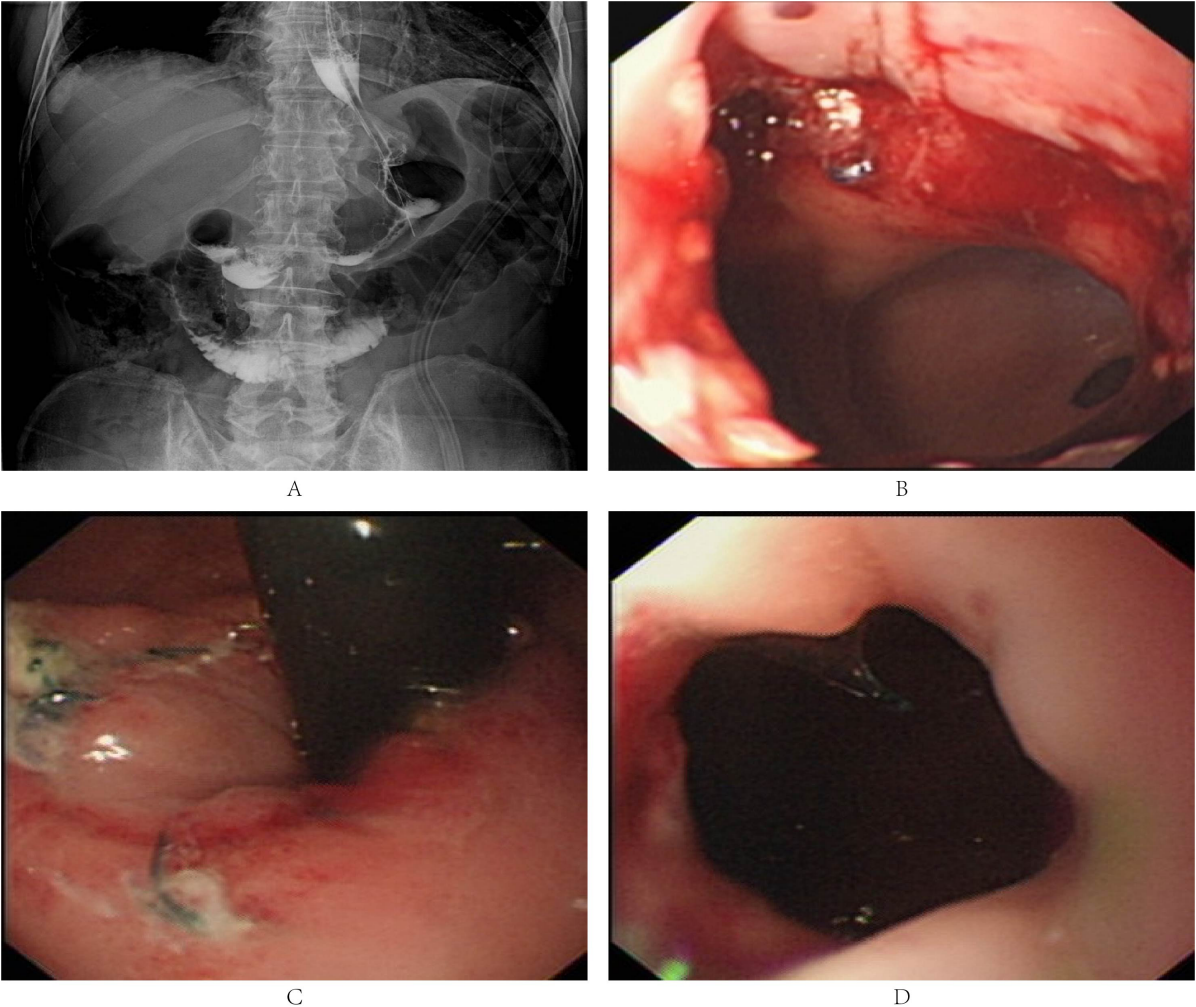
**(A)** Contrast radiography after LPG-DFT, revealing no anastomotic stenosis or anastomotic leakage. **(B-D)**. Endoscopic findings after LPG-DFT, showing no patients were suffered from reflux esophagitis (Los Angeles Grade B or more) or anastomotic stenosis.

As shown in **Table 2**, the mean operative time was 218.2 ± 45.3 minutes, with an anastomotic time of 66.2 ± 17.6 minutes. The mean estimated blood loss was 49.3 ± 31.0 mL, and no patients required conversion to open surgery or experienced intraoperative complications. Lymph node analysis revealed a mean harvest of 27.3 ± 8.9 nodes, with 0.7 ± 0.8 positive nodes per patient. All resection margins were negative. Postoperative recovery metrics included an exhaust time of 2.6 ± 0.5 days, catheter duration of 3.9 ± 0.5 days, and hospital stay of 8.6 ± 3.8 days. No major complications (Clavien-Dindo > B) or 30-day mortality occurred. Postoperative contrast examination revealed no obvious anastomotic stenosis or anastomotic leakage (**Table 2**; **Figure 3A**). Four patients received adjuvant chemotherapy based on pathological staging (pT2N1M0 or pT3N1M0).

**Table 2:** Operative results and short-term outcomes.

Indicators	Patients (*n* = 20)	
Lymph node (group)	Number of harvested (Mean)	Number of positive (Mean)
1	2.3 ± 1.6	0.2 ± 0.4
2	1.4 ± 1.2	0.1 ± 0.2
3a	4.4 ± 2.2	0.4 ± 0.7
4sa	3.9 ± 1.9	0.1 ± 0.3
4sb	3.1 ± 2.0	0
7	2.5 ± 1.8	0
8a	2.5 ± 1.4	0
9	2.0 ± 1.7	0
11p	4.4 ± 2.6	0
11d	1.4 ± 1.1 (n=15)	0 (n=15)
total	27.3 ± 8.9	0.7 ± 0.8
Operative time (min)	218.2 ± 45.3	
Anastomotic time (min)	66.2 ± 17.6	
Estimated Blood loss (ml)	49.3 ± 31.0	
Median	39.5 [20-150]	
Pathological stage		
	T1N0	5
	T1N1	5
	T2N0	6
	T2N1	3
	T3N1	1
Differentiation		
	Moderately differentiated	16 (80%)
	Well differentiated	4 (20%)
Adjuvant chemotherapy	4 (20%)	
Positive margin	0	
Length of stay (days)	8.6 ± 3.8	
Median	7 [5-20]	
Exhaust time (days)	2.6 ± 0.5	
Median	3 [2-3]	
Duration of catheter (days)	3.9 + 0.5	
Median	4 [3-5]	
Complications (≥one grade 3–4 complication), n (%)	3 (15%)	
	Major bleeding	0
	Intra-abdominal abscess	0
	Ileus	1 (5%)
	Anastomotic leakage	0
	Anastomotic stenosis	0
	Wound infection	1 (5%)
	Lung infection	1 (5%)
	Deep venous thrombosis	0
30-days mortality	0	

Data of continuous variables were presented as mean ± standard deviation and median[minimum, maximum]. Data of categorical variables were presented of absolute number of patients (%).

### Postoperative function outcomes

No patient were suffered from reflux esophagitis (Los Angeles Grade B or more) 12 months after surgery which assessed by endoscopy (**Table 3**; **Figures 3B–D**). Subsequently, no anastomotic stenosis was detected on endoscopic examination (**Table 3**; **Figures 3B–D**). Body weight and nutritional levels showed a slight decrease one year after surgery, but there was no significant difference compared to preoperative levels (**Table 3**).

**Table 3:** Nutrition status.

Indicators	Preoperative	3 months	P value	1 year	P value
Reflux esophagitis			1.000#		1.000#
No	20	19		19	
LA-a	0	1		1	
LA-b or more	0	0		0	
Anastomotic stenosis	/	0		0	
Weight (kg)	61.0 ± 9.6	58.4 ± 9.4	0.000*	60.4 ± 10.4	0.233*
Albumin (g/L)	42.5 ± 2.8	38.8 ± 3.8	0.008*	41.5 ± 3.9	0.110*
Prealbumin (mg/dL)	24.0 ± 3.5	21.4 ± 3.4	0.027*	23.3 ± 3.1	0.018*
Lymphocyte count (10^9^/L)	1.9 ± 0.7	1.8 ± 0.5	0.000*	1.9 ± 0.6	0.239*
Prognostic nutritional index	52.2 ± 4.5	47.6 ± 5.1	0.000*	50.9 ± 4.7	0.074*

#Fisher’s exact test.

*Pair sample test was used and compared with preoperative items.

**Figure 4:**
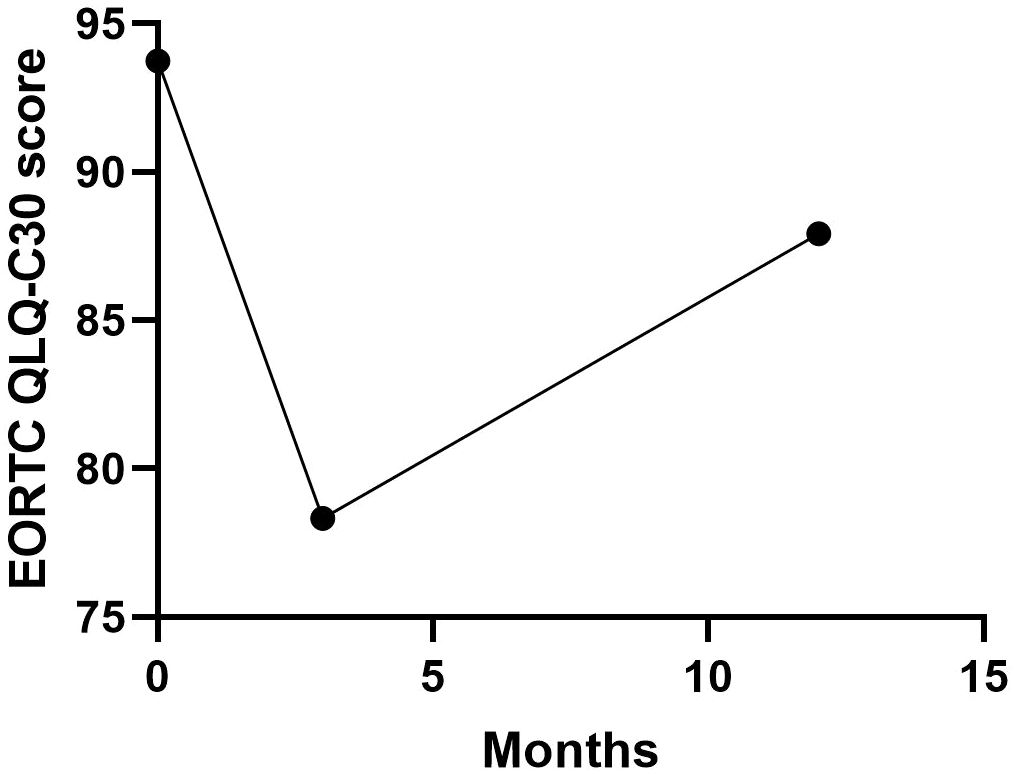
Changes in EORTC QLQ-C30 global health status. The score was not different between baseline and 1 years after surgery.

**Figure 5:**
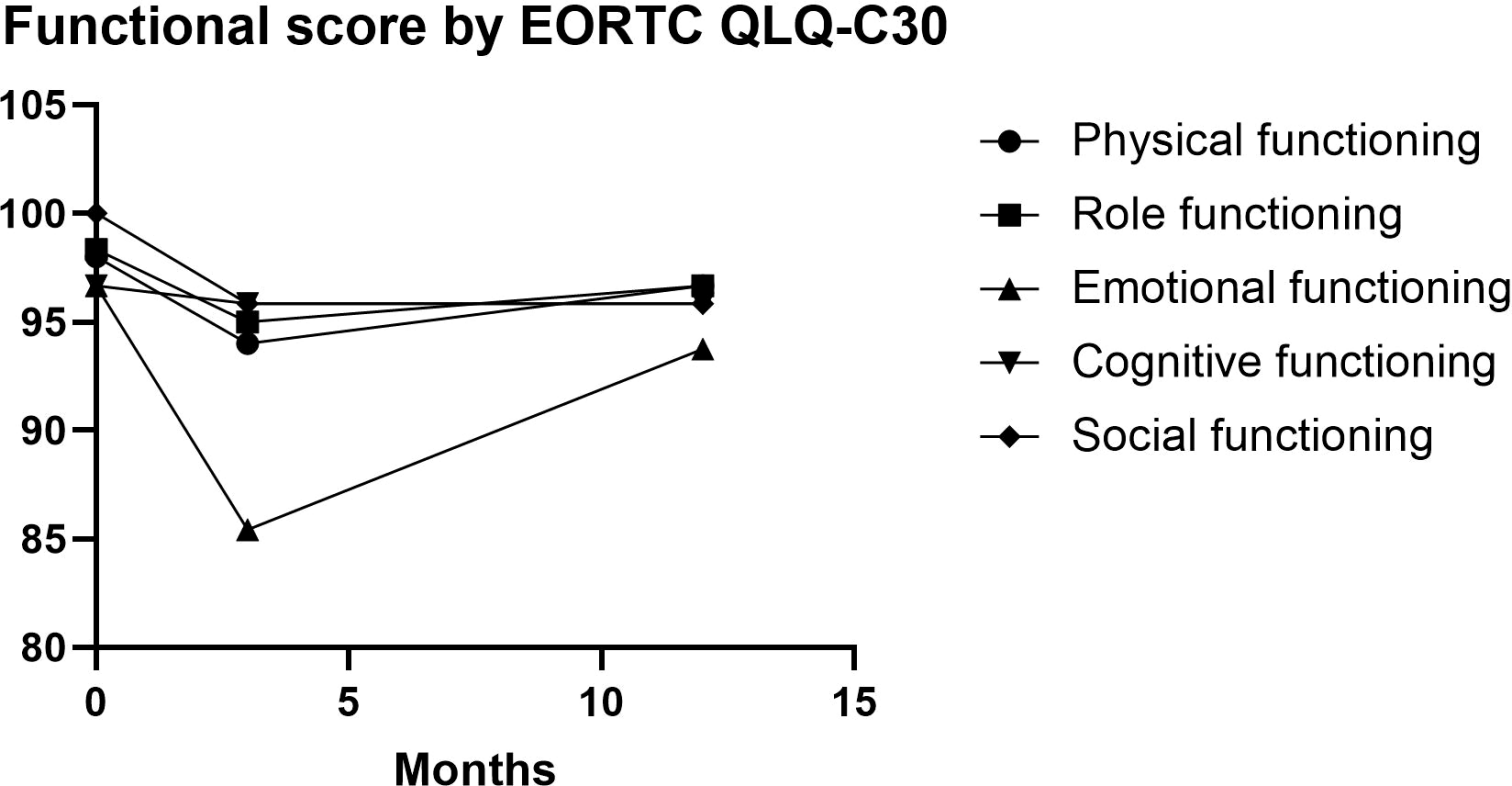
Changes in EORTC QLQ-C30 functional scales. Most scores displayed an initial postoperative decline followed by gradual recovery to baseline levels by 1 year.

The QoL was assessed using the QLQ-C30 and QLQ-STO22 score in 20 patients pre-operatively and at 3 months, 1 years postoperatively. As demonstrated in **Table 4**, the global health status scores from the EORTC QLQ-C30 questionnaire exhibited statistically significant differences when comparing preoperative baselines with 3-month postoperative assessments (p=0.001). While clinical improvement was noted at the 1-year follow-up, the scores showed no significant difference relative to preoperative values (p=0.104, **Figure 4**). Regarding functional scales, most domains displayed an initial postoperative decline followed by gradual recovery to baseline levels by 1 year (**Figure 5**). In terms of symptom scales, none of the parameters demonstrated significant worsening at the 1-year evaluation compared to preoperative measurements. The longitudinal trajectory analysis revealed a characteristic pattern of initial deterioration followed by subsequent improvement throughout the follow-up period.

**Table 4:** EORTC QLQ-C30.

Item	Preoperative	3 months	P value#	1 year	P value#
Physical functioning	98.0 ± 6.5	94.0 ± 10.4	0.036	96.7 ± 8.2	0.104
Role functioning	98.3 ± 7.5	95.0 ± 9.5	0.042	96.7 ± 8.7	0.163
Emotional functioning	96.7 ± 5.0	85.4 ± 16.6	0.004	93.8 ± 8.1	**0.015**
Cognitive functioning	96.7 ± 8.7	95.8 ± 11.9	0.330	95.8 ± 11.9	0.330
Social functioning	100.00 ± 0.00	95.8 ± 9.2	0.056	95.8 ± 9.2	0.056
Fatigue	4.4 ± 10.5	12.8 ± 17.4	0.096	7.2 ± 12.1	0.437
Nausea and vomiting	0.0 ± 0.0	2.5 ± 6.1	0.083	2.5 ± 6.1	0.083
Pain	0.8 ± 3.7	2.5 ± 6.1	0.330	1.7 ± 5.1	0.577
Dyspnea	0.0 ± 0.0	0.0 ± 0.0	1.000	0.0 ± 0.0	1.000
Insomnia	8.3 ± 14.8	10.0 ± 15.7	0.330	10.0 ± 15.7	0.330
Appetite loss	0.0 ± 0.0	10.0 ± 26.7	0.110	1.7 ± 7.5	0.330
Constipation	10.0 ± 21.9	15.0 ± 25.3	0.186	15.0 ± 25.3	0.186
Diarrhea	0.0 ± 0.0	0.0 ± 0.0	1.000	0.0 ± 0.0	1.000
Financial difficulties	21.7 ± 27.1	46.7 ± 34.9	0.004	28.3 ± 29.2	**0.035**
Global health status	93.8 ± 12.4	78.3 ± 20.7	0.001	87.9 ± 16.1	0.104

#Pair sample test was used and compared with preoperative items.

Bold formatting indicates p < 0.05.

The EORTC QLQ-STO22 scores demonstrated a similar trend (**Table 5**; **Figure 6**). While postoperative increases were observed in dysphagia, pain, reflux symptoms, dietary restrictions, anxiety, and body image scores at the 3-month follow-up, these parameters subsequently decreased by the 1-year postoperative assessment. Notably, no statistically significant differences were detected between preoperative baseline values and 1-year postoperative scores for these domains.

**Table 5:** EORTC QLQ-STO22.

Item	Preoperative	3 months	P value#	1 year	P value#
Dysphagia	0.6 ± 2.9	5.0 ± 9.7	0.072	1.7 ± 5.4	0.428
Pain	1.3 ± 5.6	4.6 ± 10.3	0.072	2.5 ± 8.2	0.186
Reflux	0.0 ± 0.0	1.7 ± 4.1	0.083	1.1 ± 3.4	0.163
Eating restriction	0.8 ± 3.7	2.1 ± 6.0	0.083	1.7 ± 4.4	0.163
Anxiety	7.8 ± 16.6	22.2 ± 20.4	0.010	11.1 ± 15.7	0.249
Having a dry mouth	0.0 ± 0.0	0.0 ± 0.0	1.000	0.0 ± 0.0	1.000
Taste	0.0 ± 0.0	0.0 ± 0.0	1.000	0.0 ± 0.0	1.000
Body image	0.0 ± 0.0	8.3 ± 21.3	0.096	3.3 ± 10.3	0.163
Hair loss	0.0 ± 0.0	0.0 ± 0.0	1.000	0.0 ± 0.0	1.000

#Pair sample test was used and compared with preoperative items.

**Figure 6:**
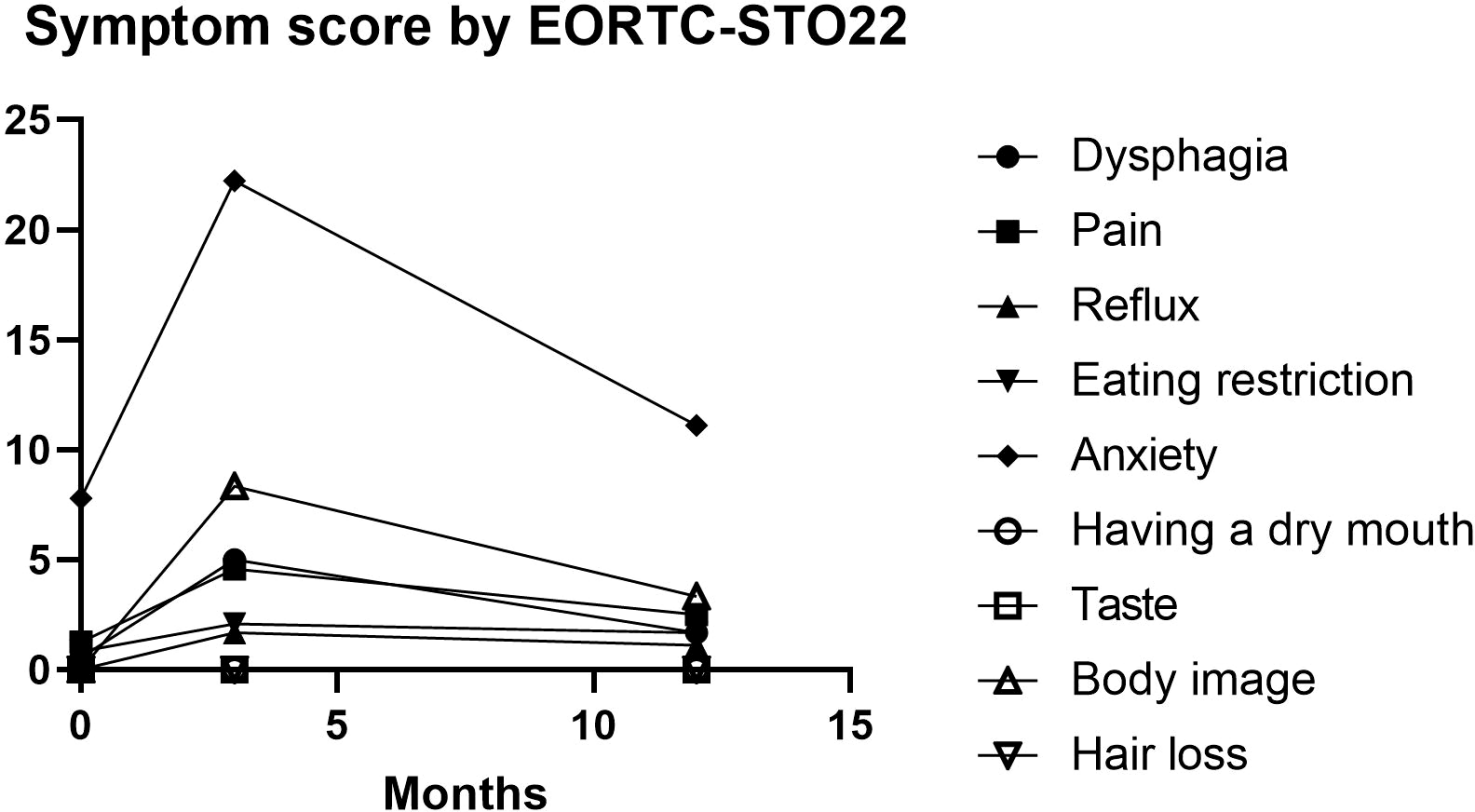
Changes in EORTC-STO22. Most scores initially increased but subsequently decreased by the 1-year postoperative assessment.

## Discussion

Although there has been a global decline in the incidence of gastric cancer overall, rates of proximal gastric cancer are rising, particularly in China (4–7). This necessitates the use of optimal surgical resection and reconstruction methods to improve patient prognosis (8).

For proximal early gastric cancer, surgeons may choose between radical TG or PG (29). The JCOG1401 trial demonstrated laparoscopic PG is safe and effective compared to laparoscopic TG (10). Although TG reduces gastroesophageal reflux risk via extensive lymph node resection, it results in permanent loss of the stomach’s storage, mechanical grinding, and secretion functions, impairs postoperative endoscopic feasibility, and can lead to malnutrition (e.g., weight loss, anemia, diarrhea, dumping syndrome) (30, 31). Conversely, PG offers advantages in preserving gastrointestinal function and nutritional status compared to TG (18, 19). However, PG’s prevalent use is limited by its association with reflux esophagitis. Therefore, developing superior reconstruction methods to decrease PG-related complications is crucial for establishing PG as the preferred treatment for proximal early gastric cancer.

DFT, an esophagogastric reconstruction method following proximal gastrectomy (PG), was first utilized by Kamikawa in 1998 for proximal gastric ulcers. Since 2016, DFT has been applied to laparoscopic proximal gastrectomy (LPG) for treating proximal gastric cancer (20, 21). Yoshiaki Shoji confirmed the efficacy of DFT in this laparoscopic context for proximal gastric cancer (32). Previous reports indicate that LPG with DFT is superior to LTG-RY in maintaining postoperative nutrition and reducing the incidence of reflux esophagitis; consequently, it is proposed as a potential first-line option for proximal early gastric cancer (13, 14). However, comparative studies of LPG-DFT and LTG-RY for proximal early gastric cancer, both within China and internationally, remain predominantly retrospective, and there is a critical lack of randomized controlled trials to establish high-grade evidence.

For this reason, we conducted a prospective, single-arm clinical trial of LPG-DFT, which is the first clinical study on LPG-DFT in China. The objective of this trial is to provide rigorous evidence to ascertain whether LPG-DFT offers additional benefits in the treatment of patients with proximal early gastric cancer. It is anticipated that this approach will become the new standard for the surgical management of proximal early gastric cancer.

We prospectively enrolled 20 patients to evaluate postoperative quality of life following LPG-DFT through serial gastroscopy and standardized assessments (EORTC-QLQ-STO22/C30), with results demonstrating no cases of Los Angeles Grade B+ reflux esophagitis at 12 months. Body weight and nutritional levels showed a slight decrease one year after surgery, but there was no significant difference compared to preoperative levels. Tomoko Tsumura et al. demonstrated that patients undergoing LTG-RY exhibited sustained reductions in body weight and nutritional levels one year after surgery, with 6% developing postoperative Grade C reflux esophagitis (13). Therefore, our study has demonstrated the feasibility and superiority of LPG-DFT in reducing the incidence of postoperative reflux esophagitis and improving nutritional status in patients undergoing proximal gastrectomy.

The QLQ-C30 revealed transient declines in global health status immediately post-surgery that normalized to preoperative baselines by 1 year, while physical/role/social functioning showed temporary 3-month decreases before returning to baseline levels. Acute postoperative increases in fatigue, pain, appetite loss, insomnia, and financial burden were observed, all of which significantly improved beyond baseline by 12 months, indicating enhanced patient comfort.

In EORTC STO22, scores for dysphagia, pain, reflux symptoms, dietary restrictions, anxiety and body image were significantly higher immediately after surgery than at baseline. These scores decreased significantly within 12 months of follow-up. Notably, no statistically significant differences were detected between preoperative baseline values and one-year postoperative scores for these domains. Scores for dry mouth, taste and hair loss remained unchanged during follow-up, indicating an excellent clinical outcome in terms of QOL after LPG-DFT. A longitudinal evaluation revealed a consistent pattern of a temporary postoperative decline in all measured parameters, followed by a gradual improvement to baseline levels, which ultimately demonstrated the capacity of LPG-DFT to deliver long-term improvements in quality of life.

Compared to total gastrectomy, the RCT study by Nicole van der Wijnen et al. showed that, regardless of open total gastrectomy or laparoscopic total gastrectomy, most quality of life modules (QLQ-C30 and STO22 scores) significantly decreased one year postoperatively compared to preoperative levels, and functional levels only recovered to about 80% of preoperative levels (33). Compared with our study, the functional recovery after total gastrectomy is inferior to that after LPG-DFT. This demonstrates that LPG-DFT is effective in improving postoperative function and quality of life in patients with proximal early gastric cancer.

Our study also has several limitations. First, the small sample size may introduce bias into the results. Second, due to the limited 1-year follow-up period, we remain unable to fully confirm the impact of LPG-DFT on long-term oncological survival outcomes. Third, as this was a single-arm study, the advantages of LPG-DFT compared to LTG-RY could not be evaluated in a head-to-head manner.

Despite the limitation, this study has showed the excellent maintenance of QOL after LPG-DFT for early proximal gastric cancer, and the strength of this study is the first prospective report about the LPG-DFT for early proximal gastric cancer in China. Further studies are needed to compare the differences of QOL between LPG-DFT and LTG-RY. Other reconstruction methods will also need to be compared with DFT.

## Conclusions

This is the first prospective study in China investigating the use of LPG-DFT for early proximal gastric cancer. The results demonstrated that LPG-DFT can improve postoperative quality of life, nutritional status, and functional status. This provides evidence-based medical evidence for the application of LPG-DFT in early proximal gastric cancer.

## Data Availability

The original contributions presented in the study are included in the article/**Supplementary Material**. Further inquiries can be directed to the corresponding authors.
